# Pyorrhœa Alveolaris

**Published:** 1882-02

**Authors:** A. O. Rawls


### ARTICLE II.

### *Pyorrhœa Alveolaris.*

BY A. O. RAWLS, D. D. S.

[Report on Pathology, to the Kentucky State Dental Society.]

The conflicting opinions relative to the causes, characteristics and treatment of this peculiar disease, are quite sufficient to convince us of the fallibility of man's judgment. They teach us that truths, whether great or small, are often hidden in the rubbish of old fogyism, or hampered by the enthusiastic misconceptions of men; therefore, in presenting a report upon this subject, I cannot but recognize the probability of error in some of my statements.

When some fifteen years ago my attention was first directed to this disease as being of a peculiar nature, I at once associated it with purely local causes, looking upon it as an inflammatory condition resulting from the disposition of salivary calculus; but after considerable treatment, based on this conviction, had yielded no very marked curative results, I naturally began an investigation in search of other causes, and to-day I present you a report of the investigation in as condensed form as possible.

*Etiology.*—The etiology of this disease is a study in itself. It is based, primarily, upon systemic conditions, and to my mind is one of the strongest proofs that certain medicinal agents, as well as custom, habit, environment, and diet, change or modify previously existing types of anatomical elements. It is a recognized fact that remedial *agents*, as well as other above mentioned influences, impress the system intensely or lightly according to the degrees of material susceptibility, accordingly as vital resistance is weak or strong, that is proportionately as chemical affinities are sharp, complete and abundant, or passive, incomplete and few in numbers.

Accepting, then, the foregoing proposition, though some may doubt its correctness, we will at once proceed to notice the causes productive of this troublesome malady.

First, they may conveniently be classified as of a two fold nature, namely, hereditary and exciting, and acquired and exciting. The hereditary and acquired conditions, though proceeding from the same primary source and alike in their tendencies, may differ in degree, but both are, nevertheless, predispositions. The exciting causes are various though generally consisting of deposits of lime salts and particles of food about the margin of the gums.

*Heredity.*—As first stated, the causes of heredity, and those acquired, are primarily from the same source, so that what I may aver of the former is, to more or less extent, true of the latter. The predisposing and active causes, transmitted from one generation to another, according to the physical and chemical laws involved in the reproduction of elementary types, are few, and consist principally of systemic conditions known as scorbutic, purpural, and mercurial.

Among the acquired causes may be mentioned all of the above, but the principal and most active cause, especially in the South and West of our country, is mercury. This has been my belief for a number of years, but was only expresed to the general profession for the first time at the meeting of the Association at Chicago in ———. At that meeting I also stated that a salt meat diet, unmixed with vegetables or acids, would cause the same, or at least such local manifestations as are present in all cases of pyorrhœa alveolaris. This opinion I still maintain, especially since I have observed that few, if any, persons once afflicted with scurvy, ever escape these oral lesions. Holding this view of the primary cause of this disease, and noticing the similarity of symptoms concomitant with both systemic, coneitions, viz: the mercurial and scorputic, I was led to investigate and search for some element or compound common to both cases of the foregoing disposition to whose actions we might, with some certainty, ascribe the oral lesions so peculiar to both maladies. Thus far I have but imperfectly concluded my labors, yet I think my observations have resulted in establishing facts sufficient to justify my statements at Chicago, and to warrant further investigation based upon theories there promulgated by Dr. Rehwinkel and myself.

One of the diseases with which we associate *Pyorrhœa Alveolaris*, viz: that of scurvy, remains yet somewhat mysterious in its causes; at least, there seems to exist a wide divergence of opinion among pathologists regarding its etiology.

It was a common opinion of general practitioners for many years that a diet of salt meat, or any exclusive salt diet, would induce scurvy; and while this is true to some extent, it is also true that scurvy can result, and has resulted from an exclusive fresh meat diet.

No matter from what source the disease arises, we know that there co-exists an impoverished condition of the blood. Dr. Wm. Headland, in speaking of the condition of the blood in this disorder, says it is deficient in albumen, fibrin, and blood corpuscles, but has a surplus of alkaline salts.

Dr. Ward denies the deficiency of fibrin and regards the diminution of blood corpuscles as the essence of the disease. Dr. Headland believes that the increase of alkaline salts, no matter by what means the increase is produced, is to be regarded as the only recognizable *materia morbosa.* Dr. A. B. Garrod held to the opinion that scurvy is attributable to a deficiency in the system of the salts of potash. So we here have opinions diametrically opposed to each other.

This much, then, for the conditions of the blood in scurvy. The effects of mercury upon the blood and general system are pretty thoroughly understood, though its *modus operandi* is still somewhat of a mystery. I think it has been sufficiently proven that substances, to enter the circulation through any of the tissues, either as food or medicaments, must first be dissolved in some one of the animal fluids, or in solution before they are introduced.

Mercury is no exception; for its introduction to the circulation, it must be finely divided, and it seems so finely divided that its presence, in a reguline state, is seldom, if ever, found in the circulation or secretions. Indeed, many of our best authorities state that its action depends upon a change of the metal into an oxide or chloride of mercury. It is patent to all that this substance, in some shape, produces a rapid and marked effect upon the quantity and physiological characteristics of the anatomical and chemical elements of the blood. According to Dr. Wright, Mr. Smith and Dr. Headland, where the system is mercurialized, the blood, by some destructive agency, is deprived of one-third of its fibrin, one-seventh of its albumen, one-sixth or more of its globules, and at the same time is loaded with a fetid matter, the product of decomposition. Herein, then, we have an impoverished condition of the blood to some extent, and that not inconsiderable, similar to that existing in scorbutic patients. It may, and with correctness, be said that like disturbances of the blood exist in other cachexias and dyscrasias, for example, scrofula, syphilis, purpuxa, etc., but the expressions of these latter systemic impressions seem to differ widely from those of the former, as they are localized in the oral tissues. It is true that any cachexia, any dyscrasia, any impoverished condition of the blood, from any source—indeed, any weakening of the chemical affinities peculiar to the normal type of the person, will tend not only to weaken the powers of physiological assimilation, but tissues already formed, and the protoplasm for reformation of new tissue; but they are surely some morbid agencies, or some morbid agent, peculiar and common to ptyalized and scorbutic patients, aside from those existing and producing similar systemic impressions, and not the same local expressions. It may be said of scurvy that the condition is owing to the deficiency of certain articles of diet, rather than to a surplus of other and different diet; but a patient can be mercurialized, no matter of what character the diet be.

And now, gentlemen, I must beg your condolence, as well as your pardon, in pressing upon you my peculiar views relative to what I conceive to be an active principle in the causation of pyorrhœa alveolaris from both systemic diseases just mentioned. The great majority of cases of scurvy from which pyorrhœa alveolaris arises, comes from a comparatively exclusive diet of salt ood, therefore salt, or some compound of one of its elements, is an active principle. Knowing that scurvy may be resultant from other exclusive diet, or from mixed diet, does not, of necessity, raise any doubt in my mind as to the probability of the formation, or rather existence of this same elementary active agency in the same undue proportion as would occur in the foregoing instance. In ptyalism we have but one element of the mercury which caused it, which is common to salt, viz; chlorine. It may be said that ptyalism is produced with metallic mercury, or with compounds of the same not containing chlorine; but some of our best authorities claim that mercury, upon entering the system, is changed before any, but a mechanical effect is produced, and that this change consists in its being either oxidized, or, as is more frequently the case, chloridized; and since the proto-chloride of mercury is not soluble in water, it must be soluble in some of the animal fluids, and since in the animal fluids and tissues, mercury is much more frequently found in the shape of the bichloride than the protochloride, we again have the evidence of a surplus of chloride.

I admit that the foregoing statement, *i. e.*, that the chloride is the combination of mercury oftenest found in the tissues of mercurialized patients, is contrary to the opinion of some of our authorities and is not supported by absolute proof; but, be that as it may, we know that the chlorides of mercury are in these cases found in proportion to the degree of the mercurial impression, all things else being equal. Now it is not contended that chlorine, *per se*, is the *materies morbi*, for it seems to me evident that the general system can and has, aided and unaided by remedial agents, rid itself of any undue proportion of this element, and after such elimination had taken place there still existed a peculiar condition of the body which rendered it susceptible to the disease in question. I am, therefore, forced to the conclusion that chlorine in some of its combinations, is inducive of a radical change in the chemical affinities existing between the circulation and nutrient fluids and the tissues they nourish. So, accordingly, not only the chemical constituents, but to more or less extent, the physical types of the anatomical elements are to a degree permanently changed, and from this systemic impression, we have the localized expression at points favoring the action of exciting causes.

I am aware that arguments have been adduced to support the theory that the susceptibility to this disease could arise from any constitutional condition which weakens the integrity of the tissues—thus, that it might arise from a tubercular diathesis, scrofula, syphilis, etc. ; but, so far as I have been able to observe, such is not the case, unless these diseases have been supplemented by a mercurial or scorbutic impression, which is at times the case. It may also be said that the very element to whose influence I ascribe the changes productive of the disposition to Pyorrhœa Alveolaris, is reckoned of much value in the curative treatment of tuberculosis and scrofula.

Thus, it is said that persons working in bleaching factories, where they are continually inhaling the fumes of chlorine gas, never have consumption, and that consumptives are improved in health and cured who subject themselves to the same influences. It is equally true that cases of scrofula are benefited by a residence near, or a voyage over, salt water; but neither scrofula nor consumption has the peculiar systemic conditions which favor Pyorrhœa Alveolaris. The peculiar chemical affinities and physical types of the anatomical elements of the first, render probable a local expression within the lung tissue of the second, the grandular system ; but in the latter, always within the tissues of the jaw, and probably other parts of the bony system. The exciting causes consist chiefly in local irritants of salivary calculus, and articles of food, but any force exerted upon the periosteum or gum, to induce the separation of the latter from the neck of the tooth, may act to the same end. Thus, continous pressure upon some one or more of the front teeth, resulting from peculiar antagonism of the molar or bicuspid teeth, the loss of an antagonizing tooth, rubber or silk ligatures, and regulating appliances, all tend, more or less, to act as exciting causes. Notwithstanding, however, the presence generally of tangible exciting causes, if we will examine closely the very earliest signs and symptoms of some of these cases, we will be puzzled at the scarcity and minuteness of physical exciting causes, and in some instances fail to find even a trace of anything foreign to the parts from external sources save the saliva and such articles of pultaceous food it might carry between the already loosened gums and corresponding teeth. Neither in these, or for that matter, the majority of cases, do we find salivary nor so-called sanguinary calculus adhering to the roots, and operating as a prime mover of this peculiar morbidity. They may, indeed, be sources of intensifying expression, and continuing the action of this local expression of systemic condition, but never the prime cause.—*Ohio Jour. of Dental Science.*

